# Empyema Necessitans in the Setting of Methicillin-Susceptible *Staphylococcus aureus* Causing Pneumonia and Bacteremia

**DOI:** 10.1155/2018/4906547

**Published:** 2018-04-05

**Authors:** Sindhura Bandaru, Sukesh Manthri, Vidya Sundareshan, Vidhya Prakash

**Affiliations:** ^1^Southern Illinois University School of Medicine, Springfield, IL, USA; ^2^Saint Louis University, St. Louis, MO, USA

## Abstract

Empyema necessitans (EN) is a rare phenomenon that refers to an insidious extension of the empyema through parietal pleura and subsequent dissection into subcutaneous tissue of the chest wall. A 29-year-old man presented to the hospital with fever and chills a few days after an inadvertent needle stick while injecting heroin. His left forearm was warm with an area of fluctuance. He underwent incision and drainage of the left forearm abscess with fluid submitted for Gram stain and culture. His condition rapidly deteriorated due to sepsis, and he required transfer to the intensive care unit. A new 4 × 3 cm area over the left pectoralis muscle had become increasingly indurated, fluctuant, and erythematous. CT of the chest demonstrated extensive cavitary lung lesions and a large loculated left-sided pleural effusion with extension through the chest wall. TEE revealed a 3 cm complex lesion on the superior septal leaflet of the tricuspid valve. The patient underwent incision and drainage of the pectoralis major EN with placement of a drain. Blood and sputum cultures grew methicillin-susceptible *Staphylococcus aureus* (MSSA) at which time antibiotic therapy was tailored to oxacillin. Our case highlights a rare occurrence of EN due to MSSA in a patient with intravenous drug use (IDU) and underscores the importance of prompt diagnosis and treatment.

## 1. Introduction

Empyema is a collection of pus in the pleural space. It is commonly caused by certain bacterial pathogens and requires drainage in addition to appropriate antibiotics for definitive therapy. Empyema necessitans (EN) is a rare entity that refers to an insidious extension of the empyema through parietal pleura and dissection into the subcutaneous tissue. EN typically occurs in the setting of long-standing parapneumonic effusions, especially those that are tuberculous in nature. It rarely presents secondary to an acute bacterial infection. We report a rare case of EN due to MSSA occurring acutely in a patient with intravenous drug use (IDU).

## 2. Case Report

**Figure 1:**
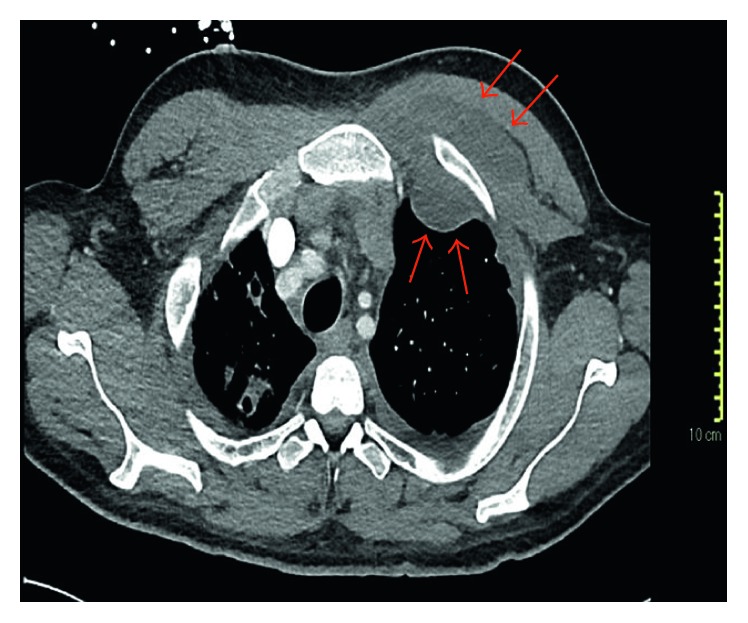
Multiple cavitary nodules within the right lung apex representing septic emboli. Bilateral pleural effusions with left-sided pleural-based focus of confluent fluid attenuation that extends through the anterior chest wall and insinuates between the pectoralis major and minor muscles representing empyema necessitans (arrows) are shown.

**Figure 2:**
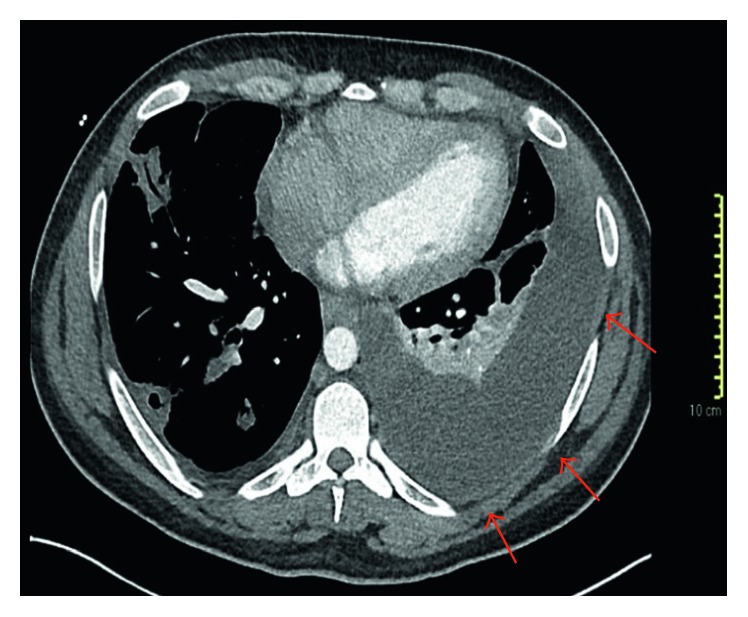
Arterial-phase axial computed tomographs of the lower thorax demonstrating a moderate left pleural effusion with associated compressive atelectasis. Peripheral cavitary pulmonary lesions and partially visualized tricuspid valve (with known vegetation) are shown.

We describe a case of a 29-year-old man with a history of IDU who presented to the hospital with fever and chills a few days after an inadvertent needle stick while injecting heroin. Avoiding medical care at first, he eventually came to the Emergency Department when he developed swelling around the punctured skin over the inferior aspect of the left cubital fossa. Upon initial evaluation, he had a temperature of 37.3°C, heart rate of 104 per minute, and respiratory rate of 38 per minute. The inferior aspect of the left cubital fossa was warm, erythematous, and with a 3.5 cm × 3.5 cm well-circumscribed area of fluctuance and induration. Laboratory evaluation revealed a hemoglobin of 10.9 gm/dl and white blood cell count of 10.6 k/cumm with a neutrophilic predominance but no immature granulocytes. His absolute neutrophil count was 9.1 k/cumm (normal 1.5–6.5 k/cumm). The comprehensive metabolic panel showed normal renal function and transaminases but elevated total bilirubin (4.5 mg/dl). His chest radiograph showed patchy infiltrates throughout the mid and upper right lung field with possible small right pleural effusion. Blood and sputum cultures were ordered, and he was started on broad-spectrum antibiotics consisting of vancomycin 1.5 gm IV every 8 hours (∼15 mg/kg), piperacillin-tazobactam 4.5 gm IV every 8 hours given as an extended infusion, and levofloxacin 750 mg IV once daily. He underwent incision and drainage of the cubital abscess. His antibiotics were tailored to oxacillin 2 gm IV every 4 hours as blood and sputum cultures revealed growth of methicillin-susceptible *Staphylococcus aureus*. On hospital day 6, his condition rapidly deteriorated due to sepsis (persistent fevers, tachycardia, and hypotension with rising white blood cell count), and a new 4 cm × 3 cm area of fluctuance was noticed on the left superior pectoralis region. He was transferred to the intensive care unit. A transesophageal echocardiogram (TEE) revealed a multilobed lesion on the superior and septal leaflets of the tricuspid valve, with lesion dimension approximating 3 cm on the superior cusp. CT of the chest with intravenous contrast demonstrated extensive bilateral cavitary lung lesions likely reflecting septic emboli, reactive mediastinal and hilar lymphadenopathy, and a large left-sided pleural effusion. A loculated component along the left upper lung insinuated through the chest wall into the left pectoralis muscle, raising the possibility of empyema necessitans (Figures 1 and 2).

Incision and drainage was performed with subsequent indwelling catheter placement for drainage of pectoralis major EN. For the left-sided pleural effusion, a chest tube was placed and it drained serosanguinous fluid. The cardiothoracic surgery service evaluated the patient and recommended ongoing antibiotic therapy with close clinical and radiographic surveillance. CT of the chest, when repeated, showed no interval change in multiple bilateral cavitary lesions, and a surveillance TEE done two weeks later identified persistent tricuspid vegetations with minimal regurgitation. As the CT of the chest was stable and the patient was clinically improving, surgery to replace the tricuspid valve was deferred with plans to treat the patient with six weeks of oxacillin.

The patient completed six weeks of intravenous oxacillin and was later started on suppressive antibiotic therapy with oral dicloxacillin 500 mg twice daily given the fact that he had high disease burden and had not undergone surgical intervention. Follow-up transesophageal echocardiogram (TEE) done soon after completing six weeks of antibiotic treatment showed that the tricuspid valve lesions had stabilized, if not minimally regressed in size, but tricuspid insufficiency had progressed to a moderate range. Magnetic resonance (MR) cardiac imaging reconfirmed sclerosis/thickening of the tricuspid valve leaflets, with moderate-to-severe tricuspid valve regurgitation. Surveillance radiography with CT of the chest showed that the cavitary lesions throughout the lungs had resolved. Unfortunately, at this point, the patient was lost to follow-up.

## 3. Discussion

Empyema necessitans (EN) refers to extension of a pleural infection out of the thorax and into the surrounding soft tissue of the chest wall and other neighboring structures. The exact pathophysiology is unclear. It may either occur in the setting of previous thoracic surgery (e.g., thoracotomy) or trauma or result from inadequate treatment of an empyema, typically occurring after a necrotizing pneumonia or pulmonary abscess [1].

The current literature notes that *Mycobacterium tuberculosis* accounts for approximately 70% of cases of EN [2]. *Actinomyces* is considered the second most common cause [3, 4]. Cases of EN due to fungal pathogens such as *Blastomyces*, *Aspergillus* species, and Mucormycosis have been described less frequently. EN due to MSSA in an immunocompetent host resulting from IDU has not previously been reported, although there have been published reports of EN due to other bacteria including MRSA, *Fusobacterium*, and *Nocardia* [5, 6].

Sharma and Blyth [7] reported an unusual case of ruptured lung abscess, complicated by a persistent air leak and EN caused by *Bacteroides* species. However, *Bacteroides* is a common cause of intrapulmonary abscess and pleural infection. Yauba et al. [8] described a pediatric case of EN due to *Proteus* species and discussed challenges in diagnosis and management as it was difficult to differentiate between tuberculous and nontuberculous effusions.

*Staphylococcus aureus* (*S. aureus*) is the most common cause of infective endocarditis (IE) in much of the developed world. Data from >70 million hospitalizations in the United States suggest that rates of *S. aureus* IE have increased significantly relative to other causes of IE [9]. *S. aureus* IE in patients with IDU often involves the tricuspid valve. Cure rates for right-sided *S. aureus* IE in IDU are high (>85%) and may be achieved with relatively short courses of either parenteral or oral treatment [10]. Complicated IE manifested by deep tissue abscesses or osteoarticular infection may require prolonged therapy. *S. aureus* IE complicated by EN is not as clearly delineated in literature, but we considered this as a complication of IE involving the tricuspid valve.

In adults, EN due to *Streptococcus pneumoniae* has rarely been reported [11], which is a similar trend for EN due to *S. aureus* infection [12].  Table 1 shows all reported cases of EN due to *S. aureus*, including pediatric cases compiled after a literature search in PubMed. Edriss and Berdine [5] described a case of EN secondary to methicillin-resistant *S. aureus* (MRSA). This patient developed a rounded painful swelling of the left upper chest after he tripped and fell. Mizell et al. [13] also described a case of EN secondary to MRSA in a patient with diabetes and cirrhosis. Unlike this case, our patient did not have any risk factors such as immunosuppression, diabetes, chest trauma, or thoracic surgery. We theorize that our patient's infection started as a skin and soft tissue infection due to MSSA in the left antecubital fossa with subsequent bacteremia and seeding of his tricuspid valve, followed by embolic phenomenon to his lungs which led to a parapneumonic effusion and ultimately direct extension through the chest wall to the pectoralis muscle, as seen in Figure 1. EN commonly spreads to the subcutaneous tissues of the chest wall and can also involve spread to other sites such as the esophagus, breast, retroperitoneal, peritoneal, pericardial, and paravertebral regions. The resultant subcutaneous abscess may eventually rupture through the skin [13].

In terms of diagnosing EN, plain radiographs are often nonspecific and at times can even be normal. At best, plain radiographs may suggest a soft tissue density in the chest wall. Chest CT is best at assessing the extent of infection out of the thoracic cavity. Chest CT will classically reveal an empyema (often a relatively well-demarcated collection) with extension through the chest wall into an extrathoracic compartment. Accompanying rib destruction may be present [5].

Management options for EN include drainage (open or closed) of the pleural space to expand the lung and mitigate risks of fibrosis. Appropriate antibiotic therapy is also a mainstay of treatment [4]. The addition of gentamicin to nafcillin or oxacillin was previously recommended for treatment of complicated right-sided IE due to MSSA. However, recent data suggest that the risks of nephrotoxicity from gentamicin outweigh the benefits of adjunctive antibiotic therapy in IE from *S. aureus*. Therapy for MSSA IE in patients unable to tolerate *β*-lactams is problematic. For patients with a well-defined history of nonanaphylactoid reactions to penicillins (e.g., simple skin rash), a first-generation cephalosporin such as cefazolin may be a reasonable choice, and vancomycin is often recommended as an alternative to *β*-lactam therapy for patients with anaphylactoid reactions. *β*-lactam allergy evaluation is recommended in cases where the nature of the allergy is not well defined, particularly due to the association of poor outcomes with vancomycin for infections due to MSSA. For patients with complications of IE such as perivalvular abscess formation and septic emboli, therapy with nafcillin or oxacillin for at least 6 weeks is warranted [18].

Our case highlights the management principles for all cases of EN, which include appropriate antibiotic therapy and management of the empyema that extends to surrounding tissue, which involves placement of an indwelling catheter and facilitates drainage and lung reexpansion.

In conclusion, EN due to *S. aureus* is an uncommon infection in healthy adults without comorbidities. EN has been associated with complications of thoracotomy, immunosuppressed status, or trauma, none of which applied to our patient. Although there have been rare reports of MRSA as a cause of EN in pediatric and adult populations, MSSA has not, to our knowledge, been reported in the literature. It is difficult to ascertain the exact reason why the pathogen in our case was MSSA as opposed to MRSA. We consider this case to have a rare presentation of EN resulting from an acute complication of MSSA pneumonia resulting from septic emboli in an immunocompetent host.

## Figures and Tables

**Table 1:** Reported cases of empyema necessitans due to *S. aureus*.

Study	Age of the patient	Isolate/organism	Risk factors	Invasive procedures	Treatment	Outcomes and complications
Stallworth et al. [14]	8 months	MRSA (blood and pleural fluid)	None	Chest tube placement	IV vancomycin for a total of 10 days, followed by oral trimethoprim-sulfamethoxazole to complete a 21-day course of antibiotics	Discharged home and on follow-up 3 weeks after discharge, the patient was afebrile and asymptomatic
Moore et al. [15]	3 months	MRSA (intraoperative cultures from the right chest wall)	None	Thoracotomy with decortication and tube thoracostomy, as well as wide drainage of the subscapular collection	IV vancomycin for a total of 14 days followed by oral linezolid for 7 days	Discharged home in stable condition. No long-term complications were reported
Mizell et al. [13]	59 years	MRSA (blood, urine, and left chest soft tissue mass)	Insulin-dependent DM, cirrhosis, heavy alcohol use, and chronic renal failure	Wedge resection of the left upper lung lobe with tube thoracostomy drainage of the left pleural space	IV vancomycin was continued for a total of 25 days, followed by a 10-day outpatient course of oral ciprofloxacin and trimethoprim-sulfamethoxazole	No long-term complications were reported
Contreras et al. [16]	19 months	MRSA (blood, pleural, and chest wall fluid)	None	Left thoracoscopic decortication and removal of fibrin-purulent exudates	Vancomycin and gentamycin were given for two weeks, followed by vancomycin alone for a total of 36 days, followed by oral clindamycin to complete treatment for osteomyelitis	Right distal femur osteomyelitis. Discharged home and at follow-up, the patient exhibited no further signs of infection
Rosebush et al. [17]	4 weeks	MRSA (right chest mass)	Exposure to a maternal breast abscess via breast-feeding	Percutaneous drainage of right posterolateral chest abscess with pigtail catheter placement	4 weeks of IV clindamycin followed by 4 weeks of oral clindamycin	Osseous involvement of the right posterolateral 9th, 10th, and 11th ribs. Discharged home. No long-term complications were reported
Edriss and Berdine [5]	60 years	MRSA in sputum and MSSA of the left hip joint aspirate	Remote history of alcohol abuse and left total hip arthroplasty	Wedge resection of the left upper lobe and treatment with IV vancomycin. For MSSA hip septic arthritis, the patient underwent total hip arthroplasty with hardware removal and antibiotic spacer implantation	Started on IV vancomycin and meropenem and discharged on 6–8 weeks of IV antibiotics	Discharged home in stable condition. No long-term complications were reported
